# Global Spread of *Trichophyton indotineae* Tracked by Mass Spectrometry Identification Network, 2022–2025

**DOI:** 10.3201/eid3209.260463

**Published:** 2026-09

**Authors:** Arnaud Jabet, Anne-Cécile Normand, Karin Amilon, Christine Schuttler, Lionel Fontao, Douglas Chan, Alicia Moreno-Sabater, Lise Kristensen, Sophie Brun, Aina Myhre, Damien Dupont, Ann Packeu, Mohammed Jami, Renaud Piarroux, MIND Study Group,, Arnaud Fekkar

**Affiliations:** Hôpital Universitaire Pitié Salpêtrière, Paris, France (A. Jabet, A.-C. Normand, M. Jami, R. Piarroux, A. Fekkar); Karolinska University Hospital, Stockholm, Sweden (K. Amilon); Laboratoire Bio Paris Ouest, Levallois-Perret, France (C. Schuttler); Hopitaux Universitaires de Genève, Geneva, Switzerland (L. Fontao); NG Teng Fong General Hospital & Jurong Community Hospital, Singapore (D. Chan); Sorbonne Université, CIMI-Paris, AP-HP, Service de Parasitologie-Mycologie, Hôpital Saint-Antoine, Paris (A. Moreno-Sabater, A. Fekkar); Aarhus University Hospital, Aarhus, Denmark (L. Kristensen); Service de Parasitologie-Mycologie, Hôpital Avicenne, AP-HP, Université Sorbonne Paris Nord, INSERM UMR1137 IAME, Bobigny, France (S. Brun); Oslo University Hospital, Oslo, Norway (A. Myhre); Hospices Civils de Lyon, Institut des Agents infectieux, Parasitologie Mycologie Médicale, Hôpital de la Croix-Rousse, Lyon, France (D. Dupont); Sciensano, Brussels, Belgium (A. Packeu)

**Keywords:** fungi, sexually transmitted infections, zoonoses, mass spectrometry, dermatophyte infection, fungal skin diseases, antifungal drug resistance, antimicrobial resistance, public health surveillance, France

## Abstract

*Trichophyton indotineae* is an emerging dermatophyte that is often resistant to terbinafine. We analyzed data produced by the Mass Spectrometry Identification global network. Those data revealed a 3-fold increase in *T. indotineae* identification during 2022–2025, demonstrating that this application is an effective tool for monitoring global spread of this pathogen.

Infections caused by the dermatophyte *Trichophyton indotineae* have recently emerged as a global public health concern (*1*). Although the first cases of infection by *T. indotineae* were retrospectively identified as early as 2008, beginning in 2016 dermatologists in India began to report a sharp increase in cases of extensive and difficult-to-treat dermatophytosis in their country. Those cases were later attributed to strains belonging to the *T. mentagrophytes* complex, which were frequently resistant to terbinafine (*1*). In 2020, the designation *T. indotineae* was proposed to denote these strains, although debate is ongoing about whether this clade should be considered a distinct species or merely a variety within *T. mentagrophytes* (*2*). Current alternative denominations include *T. mentagrophytes* internal transcribed spacer (ITS) genotype VIII and *T. mentagrophytes* variant. *indotineae*. *T. indotineae* infections are now being reported in many countries, mostly as individual case reports or small single-center series, illustrating the rapid international spread of this species through migration and travel. Only a few publications provide data at broader (regional or national) scales and over longer periods (*3*–*5*).

Macro-microscopic–based identification of *T. indotineae* remains challenging because of its close similarity with *T. interdigitale* and *T. mentagrophytes*. Reference identification relies on sequencing of the ITS1–5.8s–ITS2 region. Matrix-assisted laser desorption/ionization time-of-flight mass spectrometry has become established in recent years as a highly effective method for identifying fungal pathogens. The Mass Spectrometry Identification (MSI) application (https://msi.happy-dev.fr) is a free tool developed and hosted by the Parasitology-Mycology Laboratory of Hôpital Universitaire Pitié Salpêtrière (Paris, France) that enables accurate and fast identification of large numbers of fungal pathogens on the basis of their mass spectra (*6*). It includes a large number of reference spectra (17,563 spectra corresponding to 1,700 fungal species) and enables the identification of rare species. In March 2022, MSI was upgraded to identify *T. indotineae* with excellent sensitivity and specificity (>95% for both parameters) (*7*,*8*). The ability to produce a reliable identification relies on the presence of 2 pairs of peaks, the presence or absence of which enables *T. interdigitale* and *T. mentagrophytes* to be effectively distinguished (*8*). Furthermore, the widespread use of this free online application has enabled the creation of an extensive international network of laboratories with ≈450 regular users in 27 countries. In this study, we analyzed identification data generated by MSI to describe the spatial and temporal dynamics of *T. indotineae* infections.

## The Study

We analyzed all spectra identified as *T. indotineae* during March 2022–December 2025 by MSI users. We considered only first-intention identifications with a score of >20 (i.e., above the threshold used for the application for correct species identification as previously described [*9*]). When the same spectrum was uploaded multiple times, only 1 entry was retained. We addressed the bias caused by the increase in the number of users over time by normalizing the number of *T. indotineae* identifications relative to the number of identifications for the genus *Trichophyton* spp. and for the *T. mentagrophytes* complex (i.e., *T. mentagrophytes*, *T. interdigitale*, and *T. indotineae*).

**Figure 1:**
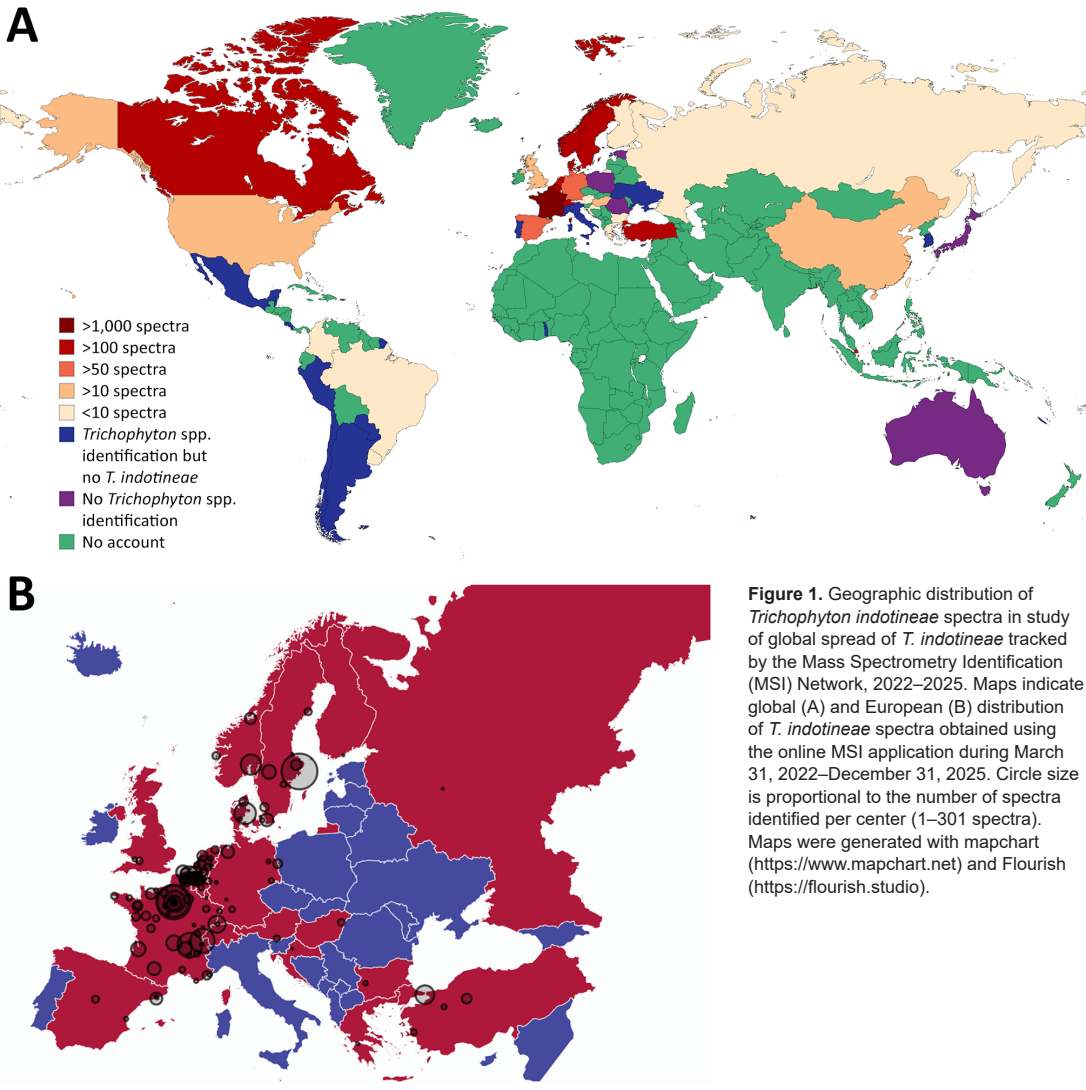
Geographic distribution of *Trichophyton indotineae* spectra in study of global spread of *T. indotineae* tracked by the Mass Spectrometry Identification (MSI) Network, 2022–2025. Maps indicate global (A) and European (B) distribution of *T. indotineae* spectra obtained using the online MSI application during March 31, 2022–December 31, 2025. Circle size is proportional to the number of spectra identified per center (1–301 spectra). Maps were generated with mapchart (https://www.mapchart.net) and Flourish (https://flourish.studio).

During the study period, 566 users from 54 countries submitted 1,657,334 spectra that led to the identification of fungal agents. Those identifications included 123,025 (7.7%) that were identified as *Trichophyton* spp. (by 316 users in 44 countries) and 3,399 (0.2%) that were identified as *T. indotineae* (by 183 users in 29 countries) (Figure 1, panel A). The number of *T. indotineae* identifications per user ranged from 1 to 299 spectra. Europe accounted for 63.8% of users, ahead of Asia (16.8%), Latin America (15.7%), North America (1.6%), Oceania (1.6%), and Africa (0.5%). As a consequence, most *T. indotineae* spectra were identified in Europe (3,040 [89.4%]). In western and northern Europe, which had the highest number of MSI users, detections were not limited to major urban centers.

**Figure 2:**
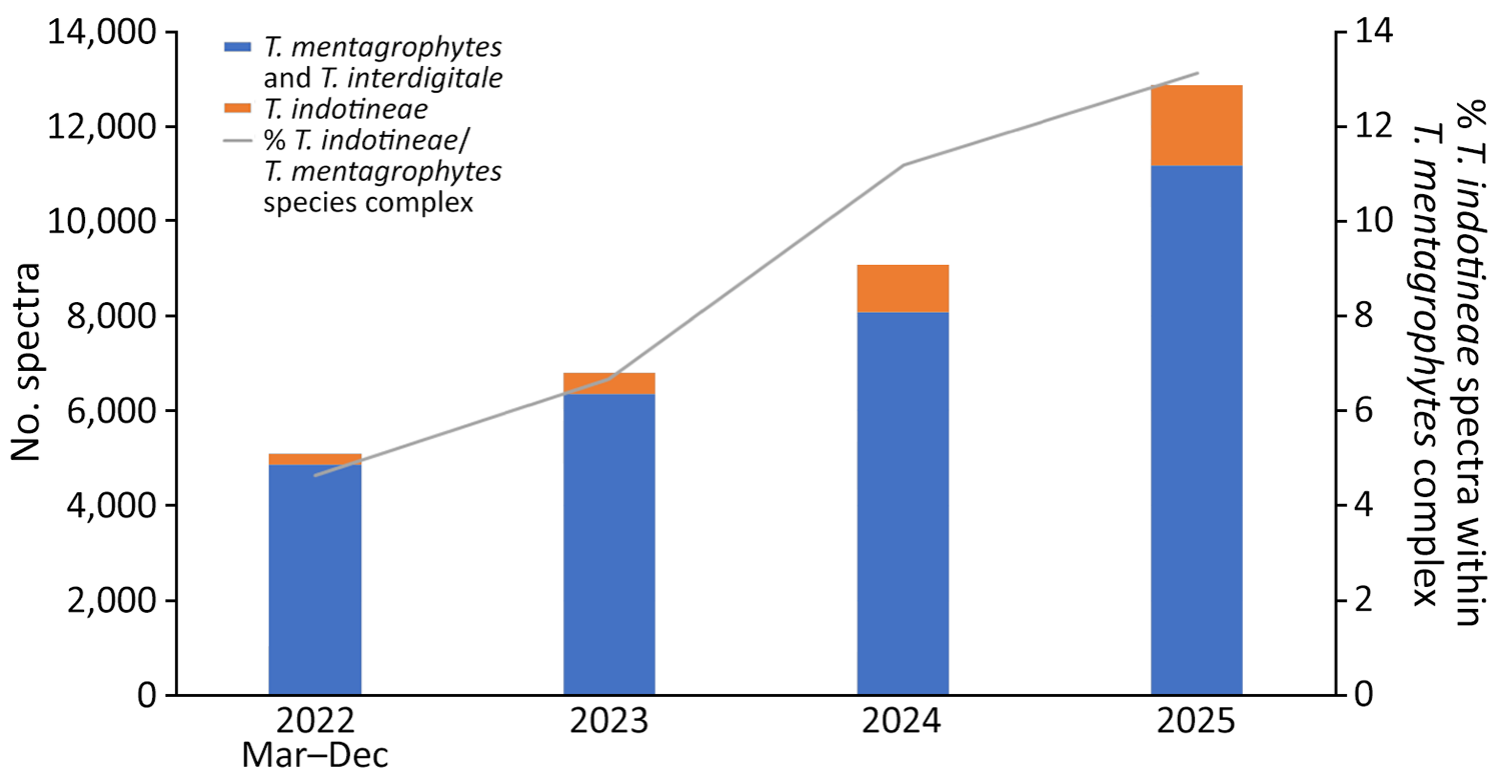
Temporal evolution of *Trichophyton indotineae* spectra identified in study of global spread of *T. indotineae* tracked by the Mass Spectrometry Identification Network, 2022–2025. Spectra were identified during March 31, 2022–December 31, 2025, and normalized to the total number of spectra within the *T. mentagrophytes* species complex (i.e., *T. mentagrophytes*, *T. interdigitale*, and *T. indotineae*).

Over the study period, the number of *T. indotineae* spectra increased continuously: from 237 in 2022 to 454 in 2023, then to 1,016 in 2024, and finally to 1,692 in 2025. At the same time, the percentage of spectra identified as *T. indotineae* within the *Trichophyton* genus increased from 1.2% to 3.6% and within the *T. mentagrophytes* complex from 4.6% to 13.1% (Figure 2). Among centers identifying *Trichophyton* spp. isolates, the percentage that detected *T. indotineae* rose from 24.7% to 46.1%.

## Conclusions

Our results reveal a marked global increase in *T. indotineae* isolation; the number of identifications rose ≈3-fold during 2022–2025. At the same time, the proportion of *T. indotineae* within the *T. mentagrophytes* complex has increased. Those findings are consistent with national or regional data that also demonstrate a temporal increase and a wide geographic distribution. Of note, the analysis of MSI data indicates *T. indotineae* was identified in countries where no cases had yet been reported, such as Bulgaria, Croatia, Colombia, Luxembourg, and Uruguay. Recent publications have already demonstrated the usefulness of MSI in identifying the first cases of *T. indotineae* in Brazil and Hungary (*10*,*11*).

The first limitation of our study is that the picture we provide of the geographic distribution of *T. indotineae* reflects both the availability of mass spectrometry devices and the use of the MSI application. Thus, no spectra were identified in Africa, where the devices are not widely available, or in well-known *T. indotineae*–endemic areas such as India and the Middle East, where few, if any, MSI users are present. Another pitfall is that the analysis is based on the number of spectra, not on the number of isolates. Inferring the number of isolates without additional data is difficult. Moreover, we do not control the conditions under which the application is used, because MSI might be used for research purposes and not exclusively for diagnostic use. Furthermore, isolates originating from a specific geographic area might be transmitted and consequently identified in another location or even another country (e.g., when isolates are sent to reference centers or used as part of research).

However, our results demonstrate that MSI can serve as a powerful interface and epidemiologic surveillance tool for emerging fungal pathogens such as *T. indotineae* or *Candida auris*. Integration of clinical and antifungal susceptibility data could enable the development of a more complete surveillance network for *T. indotineae* and bolster other initiatives, such as one supported in 2023 by the European Centre for Disease Prevention and Control that encouraged reporting of *T. indotineae* infections through EpiPulse (https://www.ecdc.europa.eu/en/publications-data/communicable-disease-threats-report-14-17-may-2023-week-20).

In non–*T. indotineae*–endemic settings, distinguishing between imported and locally acquired cases is key. The establishment of *T. indotineae* is now well documented in eastern China (*12*), and, in Germany, a growing number of infections have been reported since early 2025 among patients without recent travel history, indicating local transmission (*13*). Similarly, increased vigilance regarding transmission routes is required to improve understanding of *T*. *indotineae* dissemination mechanisms. Sexual transmission has recently been suggested as an underestimated route, which has been seen for *T. mentagrophytes* ITS-genotype VII (*14*). Because MSI is also used in veterinary laboratories, identifying host types could help monitor the zoonotic circulation of *T. indotineae*, because cases have been reported in dogs in India, Iran, and Egypt (*15*). Reliable and rapid identification of *T. indotineae* has direct clinical benefits, and real-time detection of *T. indotineae* cases could help identify patients for inclusion in clinical trials, which are needed given the frequent relapses observed after apparent cure with itraconazole therapy

In conclusion, we encourage microbiology laboratories to use MSI to identify dermatophytes for both individual and public health benefit. Health authorities should address this rapidly evolving international issue, which is associated with therapeutic challenges.
